# Mixtures of Micro and Nanoplastics and Contaminants of Emerging Concern in Environment: What We Know about Their Toxicological Effects

**DOI:** 10.3390/toxics12080589

**Published:** 2024-08-13

**Authors:** Marina Bastante-Rabadán, Karina Boltes

**Affiliations:** 1 Departamento de Química Analítica Química Física e Ingeniería Química, Universidad de Alcalá, Campus Científica Tecnológico, Ctra. A-II km 33.6, 28871 Alcalá de Henares, Madrid, Spain; marina.bastante@uah.es; 2 IMDEA Instituto Madrileño de Estudios Avanzados Water, Avda. Punto Com, 2, 28805 Alcalá de Henares, Madrid, Spain

**Keywords:** microplastics, micropollutants, combined toxicity

## Abstract

In real environments, pollutants do not occur in isolation. Instead, they can be found in complex mixtures with effects that are completely different from those of the individual components. In this review, articles from 2017 to May 2024 have been selected to provide an overview of the existing knowledge on complex mixtures between micropollutants and micro and nanoplastics in organisms in terrestrial and aquatic environments. It was found that the corresponding toxicological parameters to determine the interaction between the compounds were not calculated in most of the literature reviewed. Our analysis shows that, in aquatic environments, synergistic effects have been found more frequently than antagonistic effects. In terrestrial environments, the joint toxicological action of microplastics or nanoplastics with emerging contaminants has been less studied, but synergistic effects may also predominate. Future work should thoroughly investigate the nature of the interactions in order to properly assess the risk posed by this cocktail of compounds in ecosystems.

## 1. Introduction

Plastic is widely used in various fields such as medicine, agriculture, the agri-food industry and sectors such as packaging. It has become an essential material in everyday lives [1]. The material is used on a global scale due to its ease of use, cost-effectiveness, durability and plasticity [2]. In the 1950s, 1.5 million tons of plastic were produced; by 2016, production reached 335 million tons and, by 2021, 390 million tons [3]. This increased production has led to an increase in the amount of waste that plastics generate and, together with their mismanagement, contribute to polluting ecosystems [1]. Once plastic waste enters the environment, it undergoes physicochemical degradation processes that break it down into smaller fragments. If the fragments are smaller than 5 mm, they are called microplastics (MPs). They and their derivatives, known as nanoparticles (NPs) (0.001–0.1 µm), are a cause for concern due to their potential ecotoxicity, ability to bioaccumulate in organisms and accumulation in food chains [4].

Microplastics are classified into primary and secondary microplastics. Primary microplastics are the ones that are produced in this size range. They are added to everyday products such as exfoliating agents in cosmetics, washing powders, toothpastes, facial cleansers or as carriers for medicines. Secondary microplastics result from larger plastics degrading under environmental conditions such as sunlight, wind and water. Primary microplastics also degrade into smaller particles through photodegradation, thermal degradation or biodegradation, forming other microplastics of different sizes and shapes such as granules, pellets, fragments, microbeads, fibers, foams [5], films, ellipses, lines and flakes [6]. Polystyrene (PS), polyethylene (PE) and polypropylene (PP) microplastics are widely used in water bottles, clothing and agri-food packaging. In the textile industry, polyester (PES), polyamide (PA) and polyethylene terephthalate (PET) are used [7].

While their adverse effects are most studied in aquatic ecosystems, they are also found in sediments, soil and atmospheric environments. Wastewater treatment plants (WWTPs) are considered the main source of microplastics to aquatic ecosystems, acting as a link between those from atmospheric deposition and domestic and industrial waters [6,8].

Many published articles have focused their attention on the presence of microplastics in water over any of the other environmental matrices due to the wide range of entry sources in different ecosystems, including wastewater (domestic, industrial, sewage overflows), rain, urban and agricultural runoff, atmospheric and landfill deposition, sewage sludge, food waste, recreational fishing and tourism. The presence of microplastics in drinking water is also of concern to the scientific community and has been identified from two sources, wastewater treatment plant discharges and rainfall and urban runoff. However, microplastics have also been found in products for human consumption [9].

Plastic particles can be detected in different environments, such as the atmosphere, and their sources are diverse, including clothing materials, paints, decorations, agricultural activities, littering, construction materials, industrial emissions, marine microplastics and traffic emissions, among others. Their distribution is influenced by several factors, including population density, local infrastructure, topography and meteorological conditions such as wind, precipitation and humidity [10]. Due to their low density and small size, they can be easily transported both indoors and outdoors, but they have a significant impact on human health as they can be inhaled by us [11].

Sources of inputs to the terrestrial system are landfills, sewage, compost, irrigation, road runoff and precipitation [12] and atmospheric deposition. Sewage sludge is a major source of microplastics in soil because it is used as an agricultural amendment. As an agricultural tradition, it is common to use plastic sheeting in greenhouses to prevent adverse environmental conditions from killing the crops, but, over time, these materials degrade and break down into micro- and nanoplastics. Organic fertilizers provide a large amount of nutrients to the soil, but, due to poor residue management, these fertilizers contain microplastics. Leachate from waste dumps should also be considered [13]. In WWTPs, there are different treatments where certain processes achieve the partial removal of microplastics from wastewater but transfer them to sewage sludge [14]. As the circular economy continues to develop, sludge is very often used in agriculture as a fertilizer, thus, transporting these compounds into the terrestrial system [15].

The impact of microplastics on the terrestrial ecosystems is important because of their capability to alter soil properties, such as reduced water retention and altered porosity and soil aggregate structure. Microplastics can accumulate in plants, being a route of entry for the organisms that feed on them. Soil microbiota play a very important role in several environmental processes, such as atmospheric nitrogen fixation by colonizing the roots of some plants. They also influence various physical and chemical properties of the soil. Microplastics are causing changes in the structure and diversity of microbial communities and are creating new habitats for microorganisms that can alter the ecological functions of ecosystems [16]. The distribution of microplastics is different at different depths. Microplastics accumulate at the soil surface, and this varies according to the region of the planet, being higher in estuaries, coastal areas and agricultural, industrial and urban soils [13].

Micro- and nanoplastics are considered pollutants of emerging concern [17]. These are unknown or unrecognized compounds whose presence in the environment, in food or water, in any natural or human-made product or in any living thing, is not necessarily new, but the knowledge of the potential adverse consequences of their presence is. The surface of microplastics provides a suitable niche for forming biofilms, called “plastisphere” [18], and can act as vectors for transporting various pollutants through water to soil, especially hydrophobic organic contaminants (HOCs), including polycyclic aromatic hydrocarbons (PAHs), polychlorinated biphenyls (PCBs), perfluoroalkyl substances (PFASs), polybrominated diphenyl ethers (PBDEs) and personal care products (PCPs) [19], in addition to heavy metals of natural origin (found in the Earth’s crust) or of anthropogenic origin (produced by activities such as mining and those employed in the chemical industry). Plastics contain additives that improve the properties of plastics and can be released during their lifetime; in fact, real plastic materials were never used as pure polymers. Typically, plastics contain substances such as bisphenol A, phthalates, flame retardants and endocrine disruptors, which can be bioaccumulative and extremely toxic to bacteria and other organisms in soils and aquatic environments [20]. The sorption of organic pollutants on microplastic surfaces depends on the different characteristics and type of polymer. The sorption of pollutants can be affected because of variations in the pH, ionic strength and the content of dissolved organic matter. The physicochemical properties such as specific surface area, degree of crystallinity and pore size distribution vary substantially among different types of microplastic particles and may dominate their adsorption capacities [21].

Pollutants do not exist in isolation in the environment; they form mixtures of varying complexity, where wildlife and humans can be exposed to these “cocktails” of contaminants by different pathways. The chronic exposure of these substances at low concentrations can have a long-term effect on the organisms, highlighting the importance of rigorous assessment and monitoring [22]. Depending on the substance, this joint toxicological effect can be additive, synergistic, potentiating or even antagonistic [23]. The presence of these micropollutants in different environments is a significant cause for concern, given the potential environmental implications they may present [24,25,26,27]. Micro and nanoplastics (MNPs) can also take part in these complex mixtures. These materials enter the body by ingestion or inhalation, and those that are <20 μm can pass through the cell membrane and can cause damage to the respiratory and digestive systems as well as growth retardation, neurological damage, hormonal changes due to the presence of additives and substances adsorbed on their surface such as heavy metals, pharmaceuticals and personal hygiene products [28]. The area is currently being the subject of study [29,30,31,32], which has revealed a paucity of knowledge in the extant literature. As things stand, the current research does not allow for either the calculation of EC50 or the assessment of the type or degree of interaction, which is essential for the effective conduct of environmental risk assessment. The objective of this review is to compile the latest scientific literature on mixtures of different micropollutants together with micro- and nanoplastics in organisms in various environments, as well as to identify and describe the effects and the types of interactions between the micropollutants and the plastic particles that occur in these contexts.

## 2. Materials and Methods

The review used several databases, including the Web of Science, Scopus, Elsevier, Google Scholar, and Google, to search for papers related to the negative effects of complex mixtures with microplastics on different organisms, as well as bioremediation techniques to reduce contamination. Only scientific articles with a publication date between 2017 and 2024 since May were considered. The search was conducted using keywords such as “microplastics”, “nanoplastics”, “antibiotics”, “heavy metals”, “pesticides”, “effect”, “organisms”, “complex mixtures”, “synergy” and “antagonism”. The total number of papers that refer to microplastics and nanoplastics is 3315, of which 79 were selected for the review. For further details, please refer to the Supplementary Material (Tables S1–S4), which contains information on the number of publications in all relevant databases and research fields pertaining to these terms. Also, the interannual increment of published works on the following topics, namely, the number of publications on MPs and NPs and mixtures with pharmaceuticals or pesticides, has decreased somewhat since 2022–2021 and 2022–2023. However, the publications most frequently refer to mixtures with pharmaceuticals.

**Figure 1:**
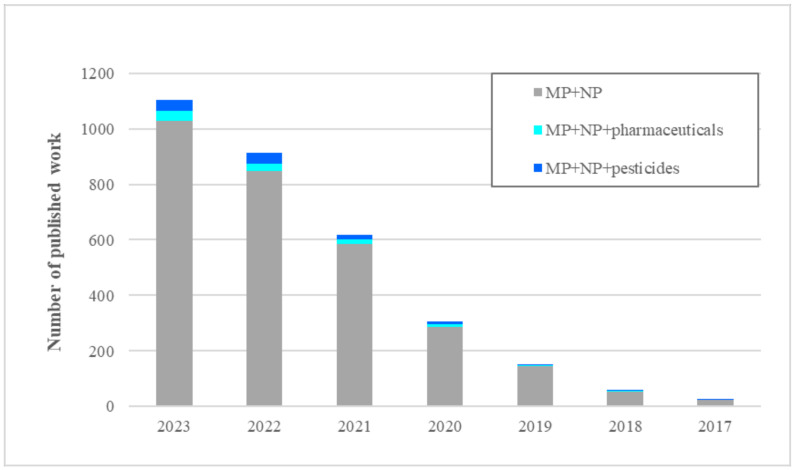
Evolution of scientific concern from 2017 to 2023 about microplastics and some of the pollutants of emerging concern. *Web of Science*, *2024*.

Figure 1 shows the evolution of the number of publications from 2017 to 2023 on micro- and nanoplastics, as well as mixing with other micropollutants. The search yield’s results for mixtures of micro and nanoplastics with pharmaceuticals and with pesticides, including mixing with other micropollutant families, were of little or no relevance until 2023. In fact, the attention of the scientific community for the combined occurrence and effect of these organic micropollutants starts to become important in 2020.

## 3. Ecotoxicity Effects of Emerging Pollutants and Micro–Nanoplastics

### 3.1. Aquatic Environment

#### 3.1.1. Mixtures Including Pharmaceutical Compounds

1. Microorganisms

Gonzalez-Pleiter et al. (2021) [33] tested the biological response of antibiotic azithromycin (AZI) and clarithromycin (CLA) sorbed on PS, PET, polylactic acid (PLA) and polyoxymethylene (POM) in freshwater algae *Anabaena* sp. *PCC7120*. The algae were grown in AA/8 þ N and buffered with HEPES 20 mM, pH 7.5. They underwent continuous shaking (135 rpm) and exposure of light of 65 mmol photons/m^2^ s illumination at 28° C. This bacterium was exposed to different treatments, microplastics (1 g in 20 mL), azithromycin (500 µg/L) and clarithromycin (1000 µm/L). These macrolides sorbed to microplastics (1 g of dried antibiotic-loaded MP in 20 mL), and the resulting concentration of the desorption experiment was, for azithromycin, in a range of 65.2–92.4 µg/mL, and for clarithromycin, 5.4–39.4 µg/L. After 72 h of exposition, an analysis of the cyanobacteria growth was taken (dry content and chlorophyll a content). The antibiotic-loaded MP was more toxic than the other treatments; the exposure had caused an inhibition of the cyanobacteria and chlorophyll content, causing a synergistic effect.

Qu et al. (2022) [34] investigated the effects of polystyrene microplastics (5 µm) and the illegal drug amphetamine on the algae *Chlorella pyrenoids*. Microalgae were exposed to different pollutants, i.e., individual exposure to amphetamine concentrations 0.01, 0.1, 1, 5 and 15 mg/L; 50 mg/L of PS MP; and the mixture of both contaminants. The algae were grown using a specific growth medium at 25 °C under 12 h light: 12 h darkness cycles, agitated three times a day. The study found that the EC50 values for amphetamine in the presence and absence of microplastics were 0.45 and 0.67 ppm, respectively. The presence of microplastics increased the toxicity of the drug, decreasing the photosynthetic pigment content by at least 10%. A significant inhibition was observed after 96 h of exposure, with 88% of cells exhibiting an inhibitory response. Furthermore, the presence of microplastics was associated with an enhanced risk of the drug, with elevated concentrations of pollutants, oxidative stress and lipid peroxidation observed. This could potentially be attributed to the adsorption of the drug into microplastic particles having a synergistic effect.

Prata et al. (2018) [35] used mixtures of microplastics ranging in size from 1 to 5 µm with the cardiac dysrhythmia agent procainamide and the antibiotic doxycycline to determine their toxicity to the marine microalgae *Tetraselmis chuii* in synthetic seawater, considering the growth rate and chlorophyll a content of algae. The algae were grown in F/2 Guillard medium, at 21 °C using a photoperiod of 16 h of light and 8 h of dark. Authors studied the mixtures of 1.5 mg/L of MP with procainamide (4, 8, 16, 32, 64, 128 and 256 mg/L) and with doxycycline (4, 8, 16, 32, 64 and 128 mg/L). After 96 h of exposure time, authors observed that microplastics alone had no significant effects on the algae growth, but the content of chlorophyll decreased in the presence of 0.9 and 2.1 mg/L of microplastics. The individual EC50 for drugs was as follows (algae growth and chlorophyll content, respectively): procainamide 104 and 143 mg/L; doxycycline 22 and 14 mg/L. Drugs caused toxicity to the organism at low concentrations, but these compounds were more toxic when mixed with microplastics, so the interaction between the pollutants are synergistic. In the case of mixtures with procainamide, the growth rate was reduced by 32 mg/L (23–73%) and, for chlorophyll a, by 8 mg/L (44–87%). Doxycycline reduced the growth rate at concentrations of 15.5 mg/L (54–100%) and chlorophyll a by 4.7 mg/L (38–98%).

2. Plants

The effects of a mixture of polystyrene nanoplastics (30 nm) and wastewater effluent from a wastewater treatment plant near Madrid, Spain, which contains numerous pharmaceutical products and drugs, on *Spirodela polyhriza* were studied by Verdú et al. (2022) [36]. The experiment was conducted under controlled conditions (25 °C; continuous 6000 lux light photoperiod), and the plant was exposed to different concentrations of nanoplastics, and wastewater dilutions, and their mixtures for a period of 72 h. Following this incubation period, the growth inhibition and fluorescence of chlorophyll in the roots, buds and leaves were measured. The EC50 for microplastics and wastewater was calculated to be 170 ± 14 mg/L and 0.94 ± 0.27, respectively. When the plant was exposed to low concentrations of the mixture, it exhibited increased growth. In contrast, the higher concentration of nanoplastics and the lower concentration of wastewater resulted in toxicity. Again, in contrast, the remaining binary mixtures facilitated enhanced plant growth compared to the individual exposure to wastewater. The nanoplastics demonstrated a capacity to adsorb the micropollutants present in the wastewater, thereby detoxifying the mixture and exerting an antagonistic effect.

In the study conducted by Mao et al. (2023) [37], the interaction between polystyrene microplastics and ciprofloxacin was investigated in two aquatic plants: *Spirodela polyrhiza* and *Lemna minor*. The plants were obtained from a lake and carefully washed with tap water to remove the sludge. They were then transplanted into glass aquaria with Hoagland nutrient solution medium in a greenhouse where the temperature was 26.3 °C with light–dark periods of 13:11 h. The plants were exposed to different concentrations of the contaminants for 15 days. The microplastics were in the range of 10–50 µm, and the concentration was 100 mg/L. The drug concentration was 2 mg/L. Following the incubation period, it was observed that the microplastic treatments did not impact the growth or specific area of the leaves of either plant. However, individual exposure to ciprofloxacin and its mixture did demonstrably affect these parameters. The effects observed on the content of photosynthetic pigments, catalase and MDA activities and soluble sugar content were species selective. Of the photosynthetic pigments in *S. polyrhiza*, a reduction in content was observed in the monoculture and in *L. minor* under mixed conditions. Individual treatments with MP did not result in a pre-sensing increase in MDA and CAT. However, when exposed to ciprofloxacin, an increase was observed. *L. minor* did not influence the contaminant mixture in terms of the amount of these enzymes. The authors identify both individual and combined contaminant interactions in the two plant species.

The study by Mao et al. (2024) [38] investigated the response of two macrophyte species, *Hydrilla verticillata* and *Elodea nuttallii*, to different sizes of polystyrene plastic (5 µm, 50 µm and 500 µm) and tetracycline. Plants were collected from a lake, carefully washed with tap water to remove sludge, and transplanted into glass aquaria with Hoagland’s nutrient medium in a greenhouse at a temperature of 31.1 ± 3 °C with light–dark photoperiods of 13:11 h for a period of 35 days. After the incubation period, samples were taken for total length, specific leaf area, branching, net fresh weight, chlorophylls a and b, soluble sugar MDA and catalase activities. The results showed that tetracycline caused a decrease in the length and number of branches in the specific area of the plants and in their net fresh weight. Treatment with MP alone also reduced these physiological characteristics, except for the 5 µm size, which stimulated growth and the content of photosynthetic pigments. For this size, the authors conclude that the effect is synergistic. In the case of the combined effect, antagonism was the result for all treatments applied.

3. Invertebrates

The aim of the study by Nobre et al. (2020) [39] was to assess the combined effect of polyethylene microspheres and the antibacterial agent triclosan on the oyster *Cassostrea brasiliana*. The study was conducted using seawater as a control test; this included seawater containing microplastics at a concentration of 250 mg/L and seawater containing 250 mg/L microplastics spiked with triclosan. Before the toxicity test, the microplastics were saturated with triclosan using at-equilibrium conditions after the adsorption experiments. Oxidative stress did not occur for 3 to 7 days of exposure because the antioxidant enzymes were not elevated, but lipid peroxidation decreased over time, possibly leading to a return to homeostasis. No DNA damage was observed either, but biochemical markers show physiological disturbances in the marine oyster related to the contact with polyethylene microspheres containing triclosan. The study did not investigate the toxicity of the mixture; however, it appears that the mixture is more toxic than the microplastic isolated. This may be attributed to an additive effect.

Nugnes et al. (2022) [40] studied the individual and combined effect of polystyrene MP, acyclovir (antiviral drug) and imidacloprid (insecticide) on *Ceriodaphnia dubia*. The organisms were exposed over the 7 days to different binary and ternary mixtures of the pesticides and microplastics to test the effects on the reproduction. They obtained the EC50 of the individual exposition: for microplastics, 1.68 μg/L, for imidaprocid, 1358 μg/L, and, for acyclovir, 0.04 μg/L. As a result, the authors reported that the antiviral compounds presented a higher toxicological effect, followed by polystyrene MP and insecticide, but the MP is the pollutant that induced higher damage in the DNA of *C. dubia*. Combining micropollutants, the authors found that a short exposure time to the mixtures induced an antagonistic genotoxicity effect on the microinvertebrates, but a long time exposure (7 days) caused an additive toxicological effect. In both cases, a negative effect occurred at very low concentrations, which was similar to the environmental concentration of the pollutants studied.

He et al. (2023) [41], in their study, performed an acute toxicity test, exposing *Daphnia magna* for 48 h to two exposure tests: MP-PS of different sizes 700 nm, 1 and 5 μm at different concentrations (0, 5, 10, 15, 20, 25 and 30 mg/L) with 50 mg/L carbamazepine; and at different concentrations of carbamazepine (0, 30, 50, 70, 90 and 120 mg/L) together with 5 mg/L of microplastics. The individual EC50 values for each toxicant were determined, with the following values observed: 88.77 mg/L for carbamazepine, 14.09 mg/L for microplastics of 1 μm, 21.66 mg/L for microplastics of 5 μm, and 24.15 mg/L for microplastics of 700 nm. The EC50 of the mixtures was also calculated, which indicated that toxicity is dependent on particle size. Furthermore, the combination of 5 μm microplastics with carbamazepine was observed to be more toxic than the other mixtures tested. Based on these observations, a 21-day generational study was conducted to investigate the impact of carbamazepine and MP on the offspring. The generations F0 and F1 were exposed to 5 μm microplastics and carbamazepine at concentrations of 5 mg/L and 5 μg/L, respectively. The breeding period was prolonged, and the number of hatchlings reduced when the concentration of microplastics was increased from 5 μg/L to 5 mg/L. The authors observed that higher concentrations of microplastics inhibited the fecundity of F0, but different patterns in the correlation between reproductive physiological performance and gene expression levels were obtained for F0 and F1 generations, with F1 responses being stronger than those of F0. It was observed that the combination with high doses of microplastics exacerbated the effects on *D. magna*, resulting in a synergistic effect between the pollutants.

4. Vertebrates

Liu et al. (2023) [42] examined the combined impact of chlortetracycline (CTC) and microplastics on *Cairina moschata*. The ducks were exposed to a concentration of 50 mg/Kg of chlortetracycline, 1000 µg/L of microplastics and a mixture of the two contaminants at the same concentration. Following the exposure period, the group exposed to microplastics exhibited intestinal inflammation and damage, as well as microbiota dysbiosis and an increase in the population of *Streptococcus* and *Helicobacter*. In the group exposed to CTC, the ducks exhibited bioaccumulation in their tissues. When the ducks were exposed to both contaminants, the microplastics alleviated the intestinal damage by regulating the gut microbiome and reduced the accumulation of the CTC in their tissues, so the interaction of the pollutants may be antagonistic.

In Banaee et al. (2023) [43], the rainbow trout (*Oncorhynchus mykiss*) was exposed to HDPE with a size of 15–25 µm and enrofloxacin in the following different concentrations: 0, 1000, and 2000 mg/Kg diet and 0, 1.35, and 2.7 mL/Kg diet, respectively, for 21 days. No mortality and no alteration in the appetite were seen in any of the organisms. Microplastics can have a synergistic effect on the drug toxicity. It has been observed that the mixture of both pollutants has more of a toxicological response than enrofloxacin alone. We also observed alterations related to triglycerides, cholesterol, glucose, urea, creatinine, total protein and albumin blood contents.

#### 3.1.2. Mixtures Including Industrial Compounds

1. Microorganisms

Microplastics can be carriers of heavy metals such as Pb, Cu, Cr, Cd, Ni, Al, Co, Zn, Mn, Fe, Ag and Hg. Liu et al. (2022) [44] reported the toxicological effect of Pb on three microplastics PP, PS and PVC and on the green algae *Chlorella vulgaris*. The algae were grown in BG11 culture media at 22 °C incubation temperature for photoperiods of 12 h in light–dark conditions. This organism was exposed through 96 h to different concentrations of Pb, i.e., 0, 50, 500 and 1000 µm/L and 0, 0.1, 0.2, 0.4 and 1 g/L of microplastics. After the individual exposition to Pb growth inhibition was observed, while the microplastics had no significant effect on algal growth, when the mixtures were carried out, a detoxifying effect was observed when the plastics were added, having an antagonistic effect, however the mixture of PP + Pb was more toxic than either substances alone. Having an antagonistic effect, however the mixture of PP + Pb was more toxic than either substances alone. This could be due to a synergistic effect.

Zhang et al. (2023) [45] investigated the combined effect of boron and three types of MP-PS on *Microcystis aeruginosa* by evaluating the chlorophyll content, photosynthetic activity and microcystin production as biological endpoints. The algae were grown (25 °C, 12:12 h light:dark cycle, 27 μmol/m^2^s) in BG11 medium. The excess of boron can be toxic for the plants, although it is an essential element for their correct development. The algae were exposed to unmodified PS, amino-modified polystyrene (PS-NH_2_), carboxyl-modified polystyrene (PS-COOH) and boron. After 96 h of exposure, the EC50 of boron was calculated, and it was 26.6 mg/L. The exposure also produced oxidative stress, had an inhibition on photosynthetic activities, and produced morphological changes in cells and an increase in toxins. The amino-modified polymer inhibited the growth of cyanobacteria, while the others did not. The authors observed that the mixture of PS-NH_2_ and boron aggravated the inhibition of chlorophyll a, and oxidative stress, and the increase in the production of microcystins, with a synergistic effect, whereas unmodified PS and PS-COOH alleviated the inhibition, with an additive effect.

The effects of gold nanoparticles (AuNPs) and microplastics on the marine microalgae *Tetraselmiis chuii* were investigated in a synthetic marine environment by Davarpanah and Guilhermino., 2019 [46]. Different exposure concentrations were tested using metal and a commercial fluorescent red microplastics of unknown composition. The concentrations tested were 0.1 g/L, 0.3 g/L and 3 mg/L for AuNPs and 0.3 g/L, 0.9 g/L and 4 mg/L, for microplastics. In addition, the following mixtures were assayed: 0.1 mg/L AuNP + 0.3 mg/L MP; 0.3 mg/L, AuNP + 0.9 mg/L MP; 3 mg/L AuNP + 4 mg/L MP. Growth inhibition was evaluated over a 96 h exposure period to determine the toxicity of the substances. The impact of the mixtures on the growth rate of algae was observed to be insignificant when compared with that of the control test. The average specific growth rate of the microalgae was reduced by 27% with the mixture of 3 mg/L AuNP + 4 mg/L MP; the other mixtures affected the growth rate, but variation was insignificant with respect to the control test, so the mixtures had a synergistic effect.

2. Plants

The individual and combined effect of polystyrene nanoplastics (80 nm) with PCB-52 on *Spirodela polyrhiza* was investigated by Pan et al. (2023) [47]. The assays were conducted within a controlled laboratory setting. The plant was cultivated under a 12-h illumination photoperiod comprising a light:dark cycle of 12:12 h, with a light intensity of 6500 lx, and a temperature of 25 °C. It was exposed to various treatments, including a control group, PCB-52 (0.1 mg/L), PS-NPs (0.5, 5, 10 and 20 mg/L) and a mixture of PCB-52 and PS-NPs (0.1 + 0.5, 0.1 + 5, 0.1 + 10 and 0.1 + 20 mg/L), for a period of 10 days. Following the exposure period, it was observed that the nanoplastics were primarily accumulated in the root and subleaf epidermal structures. The accumulation was found to be higher when both pollutants were present. The application of PS-NPs at 10 mg/L and 20 mg/L in isolation or in conjunction with PCB-52 significantly impeded the growth of the *S. polyrhiza* organism. This was accompanied by a notable reduction in the synthesis of chlorophylls a and b and an increase in oxidative stress and lipid peroxidation levels. Additionally, an osmotic imbalance was observed. The combined treatment was found to be more toxic than the individual exposures. It can be postulated, therefore, that the interaction may be synergistic.

A study by Yu et al. (2022) [48] examined the individual and combined effects of polystyrene nanoparticles (100 nm) and Bisphenol F on the macrophyte *Hydrilla verticillata*. The plant was exposed to varying concentrations of nanoplastics (1 and 10 mg/L) and Bisphenol F (10 mg/L), as well as their corresponding mixtures. This was carried out under a photosynthetic photon flux density of 80 μmol/m^2^s and a 12:12 h⁻^1^ light:dark cycle, with an exposure time of 16 days. After the exposure period, a decrease in plant size was observed at high concentrations of nanoparticles, while the lowest concentration had no effect. In contrast, the 10 mg/L mixture of each pollutant increased plant growth, indicating that the effect of the plastics was more pronounced than that of the mixture. With regard to the effects on photochemical pigments, it was observed that the plastics did not produce an inhibition of photochemical pigments. The authors found that PS-NPs alone or in combination with BPF at high concentrations can cause oxidative damage in plants. The NPs contributed to a strong inhibitory effect, which led the researchers to conclude that PS-NPs and BPF have antagonistic effects on *H. verticillate*.

The study by Li et al. (2023) [49] investigated the individual and combined effects of polystyrene nanoparticles (100 nm) and arsenic on *Myriophyllum verticillatum* L. It was carried out under controlled conditions where plants were grown in a 14:10 h light:dark cycle with a day temperature of 26 °C and a night temperature of 20 °C for 24 days. The plants were exposed to different treatments with nanoplastic concentrations of 0 mg/L, 10 mg/L and 50 mg/L and arsenic concentrations of 0 mg/L, 0.1 mg/L and 1 mg/L and their respective mixtures. After the exposure period, it was found that the high arsenic treatment reduced plant size and height by 70 and 18%, respectively, with a similar effect in the combined exposure environment. The levels of chlorophylls a and b were unaffected by the nanoplastic treatment, but changes in plant antioxidant activities were observed in combination with the higher arsenic concentration. The authors found that the interaction of the nanoplastics depended on the arsenic concentration being synergistic at a concentration of 1 mg/L and antagonistic at 0.1 mg/L.

In the study by Wang et al. (2023) [50], the individual effects of polystyrene and cadmium nanoplastics (100 nm) on *Ceratophyllum demersum* L. were investigated. The study was conducted under laboratory conditions, at a temperature of 25 °C, with a light intensity of 6000 lx. The plant was exposed to varying concentrations of nanoplastics (5 and 10 mg/L) and cadmium (0.1, 0.5 and 1 mg/L) for 14 days. The plant was grown under a 14/10 h light/dark cycle. After the exposure period, it was observed that the combined action resulted in a greater degree of growth inhibition than that observed with the individual exposures. The authors propose that the interaction of the two pollutants is synergistic. The synthesis of chlorophyll was reduced, which, in turn, caused issues with the antioxidant system of the plants.

3. Invertebrates

Jeyavani et al. (2023) [51] investigated the toxic effects of ZnO nanoparticles and polypropylene microplastics, both individually and in combination, on *Pomeacea paludosa*. The test was carried out with different treatments, where the concentration of both pollutants was 10 µm/L, in metal-free synthetic freshwater, for 28 days. Following the exposure period, samples were collected to assess oxidative stress, neurotoxicity, digestive enzyme activity and histological and genotoxic effects. The study showed that exposure to the pollutants resulted in changes in antioxidant parameters, specifically superoxide dismutase, catalase and glutathione-S-transferase enzymes; the latter two of which gradually increased to a maximum in the group exposed to both pollutants, leading to the release of oxygen free radicals. This was also associated with the increased oxidation of proteins and lipids. It was also shown that the coexistence of the two pollutants resulted in increased levels of the enzyme acetylcholine esterase, which may cause behavioral changes and mortality in organisms. There was also a reduction in the activity of digestive enzymes, in particular, esterase and alkaline phosphatase. In addition, histological analysis revealed a decrease in hematopoietic cells and a disintegration of blood vessels, digestive cells and calcium cells. Genotoxicity was also observed through DNA damage. The combined activity of both pollutants enhanced the damages in *P. paludosa*, causing a synergistic effect.

4. Vertebrates

Yan et al. (2020) [52] studied the one-month exposition of marine medaka (*Oryzias melastigma*) to polystyrene microplastics and heavy metals such as Cd, Pb and Zn. These organisms were exposed to different treatments as follows: MP-PS 100 μg/L, Cd 10 μg/L, Pb 50 μg/L, and Zn 100 μg/L and their combinations. As a result, the combination of MP-HM was the dysbiosis intestinal, changing the microbiome gut (*Burkholderiales*, *Betaproteobacteria* and *Corynebacteriales*) and activating the metabolism of the terpenoid and polyketide pathways in males. During gonadal development, exposure to both heavy metals (HMs) and a combination of microplastics and heavy metals (MPs-HMs) led to the occurrence of empty follicles (EFs) and follicular atresia (FA). Additionally, there were alterations in gene expression levels related to the hypothalamic–pituitary–gonadal (HPG) axis; the effects were caused by heavy metals, principally. The interaction of the mixture of pollutants was not studied.

The individual and combined effects of ZnO nanoparticles and polystyrene nanoplastics on juvenile *Ctenopharyngodon idella* were investigated by Estrela et al. (2021) [53]. In the study, the fish were exposed to a concentration of 760 µm/L of each contaminant for three days. After this period, the researchers conducted behavioral tests and collected data on the toxicity of the contaminants before sacrificing the animals. The study showed that exposure to the pollutants resulted in the inactivation of the organisms. It was also observed that the NPs, either alone or in combination with ZnO, stimulated antioxidant activity, but the pollutants had no effect on antioxidant activity, which was not studied but it may be an additive effect.

Most recently, Zhang et al. (2024) [54], investigated the interaction between polystyrene microplastics (10 mg/L) of two sizes (5 and 50 µm) and the polyhalogenated carbazol 3,6-dibromocarbazole (0.5 mg/L) on zebrafish embryos (*Danio rerio*) in an aquatic environment (unchlorinated tap water). The study recorded embryo hatching rates at 48, 72 and 96 h post fertilization (hpf) using an inverted microscope, as well as embryo mortality and teratogenicity at 96 hpf. After exposure for 96 hpf, it was found that the mortality and hatching of the embryos was not compromise, but the individual exposure to pollutants resulted in an increase in oxidative stress and, then, caused apoptosis. The combinate effect on the embryos significantly reduced the effects, causing an antagonistic effect.

Xu et al. (2021) [55] conducted a study on the effects of microplastics and phenanthrene on *Danio rerio*. The fish were exposed to 3 mg/L polystyrene microplastic and 0.2 mg/L phenanthrene, as well as a combination of both, for 12 and 24 days to study the accumulation, antioxidant enzymes, gene expression and gut microbiota. Consequently, it was observed that individual exposure to the pollutants results in alterations to the oxidative stress and immune system of the fish. However, the combination of both compounds exacerbates these effects, facilitating the bioaccumulation of the compound in the organisms, which, in turn, causes further oxidative stress and alters the microbiota, increasing Proteobacteria and Bacteroides. The authors may have identified a synergistic effect.

Sayed et al. (2024) [56] conducted a study investigating the effects of polystyrene nanoplastics with a size of 50 nm and a concentration of 5 mg/L, in combination with engine oil at a 1% concentration, on *Oreochromis niloticus* over a period of 15 days. The fish were subjected to a series of treatments, comprising individual exposure to each pollutant and a combination of both. A range of hematobiochemical parameters, along with pro-inflammatory cytokines and oxidative stress biomarkers, were evaluated in conjunction with a detailed examination of any histological alterations. After the exposure, groups that were exposed to engine oil and the combination with nanoparticles exhibited erratic swimming patterns, abnormal behavior, and a loss of reflexes. Alterations in all of the measured parameters were accompanied by severe necrotic and hemorrhagic lesions, as well as an inflammatory response, oxidative stress and lipid peroxidation in the combined exposure. The authors found that a synergistic effect was observed.

#### 3.1.3. Mixtures Including Agrochemical Compounds

1. Microorganisms

The toxicity of amino-modified polystyrene nanoparticles (nPS-NH2) and glypho-sate on *Microcystis aeruginosa* was investigated under laboratory conditions by Zhang et al. (2018) [57]. The cyanobacteria were grown in BG11 culture media under cool light at 25 °C. After exposure to single concentrations of glyphosate (0.5, 1, 3, 5 and 7 mg/L) and nanoplastics (3, 5, 10 and 20 mg/L), the EC50 values for 48 and 96 h were determined to be 8.1 and 6.3 mg/L, respectively. Combination treatments included single pollutants as a control, using 1 and 5 mg/L for the pesticide and 5 mg/L for the NPs and their binary mixture. The results suggest that the toxicity of a single compound was greater than that of the combined compounds. The authors found evidence of glyphosate adhesion to the nanoplastics, which, in turn, facilitated its adsorption to the algae, leading to the biomagnification of the contamination along the food chain, so the interaction may be antagonistic.

2. Plants

The study by Tang et al. (2024) [58] investigated the effects of polystyrene nanoplastics and imidacloprid on *Cyperus papyrus* L. Experiments were conducted in a greenhouse at 28 °C under controlled conditions. For this experiment, two types of polystyrene, amino-modified PS and PS-COOH at a concentration of 50 mg/L, as well as imidacloprid at a concentration of 1.0 mg/L, were used. The different treatments were applied for a period of 28 days. At the end of the exposure period, it was found that the single exposure of the amino-modified PS inhibited plant growth. Its combined effect with the pesticide reduced the fresh weight and length growth rate, as well as the chlorophyll a/b content in the plant, more than the single exposure did, which the authors describe as a synergistic effect. The antioxidant response in the plant was activated by both types of plastic in combination with the pesticide.

3. Invertebrates

Ziajahromi et al. (2019) [59] investigated the effect of PE-MP on the bifenthrin toxicity of *Chironomus tepperi* in both synthetic and riverine waters, with experiments conducted in parallel. Individual and combined 48 h exposure bioassays were performed with different concentrations of bifenthrin (nominal concentration from 0.1 to 3.2 µm/L) and 5 mg/L PE-MP in the size range 10–27 µm. After 48 h of exposure, EC50s were calculated in synthetic and river water for individual and combined exposure at 0 and 1.3 µm/L and 1.3 and 1.4 µm/L, respectively. In conclusion, bifenthrin was more toxic in synthetic water than in combination with microplastics, with an antagonistic effect, reflecting a lower survival rate in the treatment with the pesticide alone, but, in real water, the toxicity of bifenthrin was lower than the mixture, with a synergistic effect.

Zocchi et al. (2019) [60] reported that microplastics can modify the toxicity of the herbicide glyphosate of *Daphnia magna*. The authors evaluated the toxicological response of invertebrates to a mixture of PET and PA fibers (length of 10 μm and width of 2 μm); PE microbeads (1 to 10 μm of range size); and three different formulations of the herbicide, during 7 days of exposure time. After this exposure, it was observed that the PE microbeads exerted a synergistic effect in combination with two glyphosate formulations (glyphosate acid and Roundup Gran). No EC50 values were obtained. By contrast, the presence of the PE microbeads decreased the negative effect of glyphosate-IPA salt. The results on the combined toxicity are not clear and appear to be dependent on the glyphosate formulations and the polymers evaluated.

The effect of microplastics on the sensitivity of *Daphnia magna* to the insecticide deltamethrin was investigated by Felten et al. (2020) [61]. The study established different conditions, including two concentrations of the insecticide (0 and 40 ng/L) and three concentrations of microplastics (0, 1 and 10 mg/L), with the exposure of Daphnia for 21 days. In the control experiment (MP0-DM0), the survival rate was 100% over 21 days. However, in MP10-DM40, all of the animals died, and, in MP1-DM40, the survival rate was only 20%. The other mixtures maintained a high survival rate. In terms of the hatch rate, MP1-DM40 showed a delay compared to the control, with exposure to 1 mg/L MP and exposure to DM40 alone (both delayed by 1.3 days). In this test group, the number of hatchlings per survivor was reduced by 51.1% and 46%, respectively. In addition, body size was reduced by 1.14% and 2.54% compared to the single exposure treatments. The effect of both pollutants may be synergistic due to the enhancement of the toxicity of deltamethrin in the higher concentration of microplastics.

4. Vertebrates

Luo et al. (2021) [62] investigated the effects of imidacloprid exposure on adult zebrafish (*Danio rerio*) under laboratory conditions. The study examined the effects of imidacloprid at concentrations of 100 µm/L and 20 µm/L PS, as well as a combination of both, over a period of 21 days. After 21 days, the animals were euthanized, and their livers were collected for the determination of several liver parameters. These included glucose, pyruvate, total cholesterol, triglyceride, low-density lipoprotein cholesterol, superoxide dismutase and catalase activities, and glutathione and malondialdehyde levels. The results showed that, in the treatments with microplastics and pesticides in low concentrations, there was an enhanced growth inhibition, causing a synergistic effect. The exposure to combined exposure may enhance zebrafish hepatotoxicity, which is characterized by gene expression changes in glycolipid metabolism and inflammation. An inflammatory response was also observed in the animal’s liver.

Mohamed et al. (2023) [63] conducted an acute toxicity test to determine the lethal dose of the pesticide Upgrade in *Oreochromis niloticus* fish. The LD50 values of the pesticide at 48, 72 and 96 h were 56.37, 36.10 and 29.16, respectively. The highest and sub-lethal concentrations of the pesticide caused various respiratory and behavioral problems. After calculating the LD50 values, the fish were exposed to the pesticide at 2.916 mg/L, 1/10 of the 96 h LC50, and PE microplastics at a concentration of 10 mg/L for 15 days. The pesticide alone had a negative effect on the hematological and biochemical parameters compared to the microplastics alone, but, in combination, the effect was attenuated by the presence of microplastics, so the mixture had an antagonistic effect.

A review of the literature shows that mixtures of microplastics with other micropollutants are more toxic to the aquatic environment than individual pollutants. Most of the articles reviewed show synergistic interactions. In microorganisms, the combination of these pollutants inhibits their growth or promotes the production of toxins into the environment. In plants, it reduces their growth, photosynthetic pigment content and oxidative stress, but, in some cases, plastics have been shown to have an antagonistic effect, reducing the toxicity of the mixture. For invertebrates and vertebrates, synergistic effects are common, especially when microplastics are combined with heavy metals or pesticides, which increase oxidative stress and neurotoxicity. Combinations of microplastics with pharmaceuticals or industrial compounds may increase toxicity, suggesting an increased vulnerability of these organisms to combined contamination.

### 3.2. Terrestrial Environments

#### 3.2.1. Mixtures Including Pharmaceutical Compounds

1. Microorganisms

Wang et al. (2020) [64] investigated the combined effects of ciprofloxacin and PE microplastics on soil properties and microbial structures. The contents of 1% (*w*/*w*) MPs and 10 mg/Kg of ciprofloxacin were used in this study. There were different treatments as follows: CK (original soil), E (CK + 1% PE), C (CK + 10 mg/Kg ciprofloxacin) and CE (CK + 1% PE + 10 mg/Kg ciprofloxacin). During five weeks of incubation, 60 g soil samples were collected on different days. The sequencing of the 16S rRNA gene was undertaken to identify the microbial communities of the soil. This work found that the degradation of ciprofloxacin was significantly inhibited when MPs were present. The total nitrogen concentrations in the soil amended with MPs and ciprofloxacin were significantly decreased compared with that of the soil amended with ciprofloxacin alone. The high-throughput sequencing results illustrated that the combined pollution of MPs and ciprofloxacin significantly decreased the soil microbial diversity when compared with that of individual contamination. As for the community structure, the microbial compositions at the phylum level were consistent among all treatments, and the most dominant phyla were Proteobacteria, Actinobacteria and *Chloroflexi*. However, at the genus level, four genera were significantly altered in the MP-ciprofloxacin-co-amended soil; *Serratia* and *Achromobacter* were also abundant and involved in the acceleration of soil total nitrogen dissipation.

In 2023, Ya et al. [65] conducted a study on the effects of coexisting tetracycline and polyethylene microplastics on the soil microbial community. They also conducted an analysis of antibiotic resistance genes (ARGss). The resulting mixture was homogenized, and 500 g of the mixture was taken and incubated. In the study, different combinations of the original soil, soil with 10 mg/Kg tetracycline, soil with 5% *w*/*w* PE and soil with both contaminants (at the concentrations mentioned above) were mixed, and deionized water was added every week until the end of the 5-week experiment. The effects of microplastics, tetracycline and ARGs on the soil microbial community were analyzed. The results show that the combined contamination of microplastics and tetracycline significantly increased the content of solid organic carbon, while it decreased the activity of neutral phosphatase in the soil. The addition of PE microplastics (5% *w*/*w*) significantly reduced the microbial diversity of the soils according to the structural characteristics of the soil microbial community analysis. Furthermore, bacterial genera such as *Aeromicrobium*, *Rhodococcus*, *Mycobacterium* and *Intrasporangium* were more affected by the combined contamination of polyethylene microplastics and tetracycline. The AGRs showed that adding polyethylene microplastics inhibited, to some extent, the degradation of ARGs in tetracycline-contaminated soils. The combination of both pollutants had a synergistic effect, decreasing the microbial diversity of the soil.

2. Plants

Khan et al. (2023) [66] investigated the individual and combined effects of the drugs norfloxacin and sulfadiazine together with polystyrene microplastics on *Chrysanthemum coronarium* L. The study used concentrations of 50 mg/Kg norfloxacin, 10 mg/Kg sulfadiazine and 4% soil *w*/*w* microplastics. After 60 days of exposure, plants were harvested and analyzed. The study showed that the presence of microplastics increased sulfadiazine uptake, whereas norfloxacin was absorbed independently, but both contaminants accumulated and translocated in plant organs. Structural changes in leaves and roots were also examined, revealing cellular and metabolic changes resulting from both single and combined exposures. In the case of sulfadiazine, the nucleus of the cells was displaced, and, for norfloxacin, an abnormal distribution of cells was observed. Concentrations of mineral elements such as K, Fe, Mn, Mg and Zn were also shown to be affected by the presence of contaminants. In treatments with sulfadiazine, the contents of Mg and Mn increased more than the others; in contrast, the Fe content was higher in the mixture, norfloxacin and microplastic treatments. The content of K was reduced in the treatments with the presence of microplastics, and treatments with the mixture decreased the content of Zn. The authors observed that the effects were greater with the co-exposure to pollutants; this may be a synergistic effect.

3. Invertebrates

The behavioral and biochemical effects of ciprofloxacin alone and in combination with polyethylene terephthalate (PET) microplastics on *Hediste diversicolor* in synthetic seawater were investigated by Araújo et al. (2023) [67]. Two behavioral parameters, spontaneous activity and burrowing behaviors, were tested after 27 days of exposure. In the study, the ability of the organisms to travel a distance in a silicone tube was assessed. Then, the organisms were placed on a clean sediment, and the time taken for them to completely burrow into the sediment was observed. The next day, tests were carried out on the metabolic enzymes of phases I and II, examining the antioxidant defense, the oxidative damage and the acetylcholinesterase. Following on from the previous behavioral and motility study, it was observed that the groups exposed to high concentrations of microplastics took longer to move and bury themselves and chronic exposure to ciprofloxacin resulted in faster burrowing, increased some antioxidant parameters and altered metabolic activity. However, it did not cause oxidative stress or neurotoxicity. However, the mixture had a synergistic effect.

The aim of Ma et al. (2020) [68] was the evaluation of changes in the microbial community of *Enchytraeus crypticus*. The soil was contaminated with tetracycline (TC), polyamide (PA) and polyvinyl chloride (PVC) of a size of 30 µm as well as the combination of both plastics with the drug (TC-PVC and TC-PA). The microcosm was established by adding 10 adult organisms of similar size and well-developed clitellums to 30 g of soil containing 1000 mg/Kg of microplastics and 20 mg/Kg of tetracycline. After 21 days of exposure, the surviving adults were analyzed for microbial communities. The results showed that the presence of both pollutants changes the microbial community of the insect, and PA-tetracycline promoted *Firmicutes* growth, compromising the worm’s health. However, in the case of PVC-tetracycline, no alterations were observed; this may be due the different adsorption of tetracycline in its surface. The authors are not sure about the type of interaction.

Xiang et al. (2019) [69] investigated the effects of sulfamethoxazole on the gut microbiota of *Folsomia candida* and antibiotic resistance when it was combined with the polystyrene microplastics of approximately 2 μm in size. The concentration of sulfamethoxazole used in this study was 1 ppm (*w*/*v*), and the concentration of microplastics was 1% (*w*/*w*). The study included three treatments: control diet, diet containing 1% polystyrene without sulfamethoxazole and diet containing sulfamethoxazole. After 28 days of exposure, the insects were sampled for DNA, morphological parameters and isotopic fractionation (C and N). As a result, it has been observed that the loaded plastic with the drug changes the gut microbiota and may cause dysbiosis to the collembolan, so the microplastics enhance the toxic effects of the drug. Therefore, the interaction may be synergistic.

#### 3.2.2. Mixtures Including Industrial Compounds

1. Plants

The 2023 study by Han et al. [70] investigated the impact of individual and combined exposure to cadmium and polypropylene microplastics on wheat growth. The experiments were conducted with two sizes of microplastics, 50 and 100 µm, in a suspension of 500 mg/L and 40 mg/L of cadmium chloride. The time of germination, growth of the root and shoot, dry weight and antioxidant enzymes were evaluated. As a result, the rate of seed germination was found to be dependent on the mixture of pollutants present. This phenomenon occurred regarding the germination energy. The combination of both pollutants (50 µm PP) resulted in an increase in root length, while the shoot length was reduced by the exposure to the mixture (100 µm PP). Co-exposure resulted in the disruption of the regulation of peroxidase and catalase, with superoxide dismutase inhibited by the mixture of 100 µm of microplastics. The authors identified that the interaction between these contaminants under experimental conditions exhibited a dependency on the size of the microplastics, with 50 µm exhibiting antagonistic effects and 100 µm exhibiting synergistic effects in combination with cadmium.

2. Invertebrates

The effects on *Folsomia candida* to the exposure of bisphenol A, diphenhydramine and nanoplastics were studied by Barreto et al. (2023) [71]. The authors assessed an individual exposure for 28 days to different concentrations of bisphenol A and diphenhydramine at 0, 1, 10, 100 and 2000 mg/Kg of soil dry weight. After the exposure, it was observed that the higher concentrations of the pollutants produced a decrease in reproduction. The calculated EC50 values for bisphenol A and diphenhydramine were 1399.3 and 1498.2 mg/Kg. In the combined exposure, the highest concentration of bisphenol A and diphenhydramine at 2000 mg/Kg and 0.015 and 600 mg/Kg of nanoplastics was used. The authors studied endpoints such as reproduction, survival, behavior, neurotransmission and oxidative stress. As a result, the authors found that the effects of the different endpoints had different interactions.

The individual and combined effects of PE-MP and CuSO_4_ on *Pontastacus leptodactylus*, a freshwater crab, were investigated by Zeidi et al. (2023) [72]. The crabs were exposed to different treatments of CuSO_4_ (0.5 and 1 mg/L) and PE-MP (0, 0.5 and 1 mg/L) in synthetic freshwater for 28 days. At the end of the exposure time, several biomarkers associated with oxidative stress were investigated. The co-exposure of both pollutants had a synergistic effect, altering the levels of biochemical markers in the hemolymph of crabs, as well as the oxidative stress parameters and immunotoxicity parameters, resulting in a decrease in detoxification capacity.

3. Vertebrates

Salla et al. (2024) [73] evaluated the developmental toxicity of bullfrogs *Aquarana catesbeiana* on individual and combined exposures to TiO_2_ nanoparticles and PE microplastics. The embryos were exposed to microplastics and TiO_2_ at concentrations of 60 mg/L and 10 µm/L, respectively, for 96 h in FETAX medium. Developmental stages, survival rate, hatching rate and the presence of morphological abnormalities were monitored. The findings showed that plastic fragments were adhering to chorionic membrane, potentially altering embryonic osmoregulation, and increasing the incidence of morphological abnormalities. The survival and hatching rates of bullfrog embryos were significantly reduced by the combination of contaminants, the effect being most pronounced in the case of isolated nanoparticles, which agglomerated in the FETAX solution, thereby reducing their bioavailability.

#### 3.2.3. Mixtures Including Agrochemical Compounds

1. Microorganisms

The effects of combined exposure to imidacloprid and flumioxazin and microplastics from aged mulch films on soil microorganisms and elemental cycling in cotton fields were investigated by Wu et al. (2024) [74]. The plants were exposed to different concentrations of microplastics (2 *w*/*w* and 2 *w*/*w*), imidacloprid (0.5 and 5 mg/Kg) and flumioxazin (0.3 and 3 mg/Kg) that were mixed with 250 g of soil and then incubated in the dark for 56 days. Sampling took place throughout the exposure period. Compared to the exposure to microplastics alone, the study found that the presence of both pesticides had a greater inhibitory effect on the community structure. The symbiotic network of the community was affected, resulting in a 13.7% decrease in complexity for microplastics, an 11.3% decrease for pesticides and a 15.0% decrease for mixed exposure. Exposure to microplastics and pesticides had different effects on the nitrogen, carbon, and phosphorus cycles. Specifically, exposure to microplastics resulted in higher concentrations of nitrate in the soil, while the effects on the carbon and phosphorus cycles were less pronounced. However, when the exposures were combined, the effects were neutralized. The combined exposure to MPs and imidacloprid increased the genes for nitrification and denitrification, but, in the case of the flumioxazin exposure, the denitrification was decreased. The effect on the genes for element cycling was observed. In addition, the combined exposure increased the abundance of microorganisms involved in the carbon and nitrogen cycles, except for the carbon cycle after flumioxazin exposure. Microplastics alone decreased the abundance of microorganisms involved in the nitrogen and carbon cycles. The combined effects were observed to be less than that of the individual effects with, in some cases, an improvement being noted. Pesticides exerted the most significant effect, and, in some instances, this effect was found to mask the presence of microplastics, although not entirely.

2. Plants

Nie et al. (2024) [75] monitored the effects of differently loaded polystyrene microbeads (PS-COO^−^, non-modified PS and PS-NH_3_^+^) on the fate of the C14-labeled antiviral pesticide dufulin in a hydroponic tomato system over a 60-day exposure period. Both contaminants were present at concentrations of 50 mg/L of microplastics and 2 mg/L of pesticide. The presence of microplastics alone had a negative effect on the growth of the tomato plants. The control group, followed by the PS-NH_3_^+^, non-modified PS and PS-COO^−^ groups, showed the highest bioaccumulation and translocation of dufulin in tomato plants. The presence of microplastics, especially PS-COO^−^, reduced the bioavailability of dufulin in tomato plants, possibly due to the strong adsorption of DFL on PS-COO^−^ and its inhibitory effect on plant growth. In addition, PS-COO^−^ significantly reduced the concentration of dufulin in tomato plants, causing an antagonistic effect.

The bioaccumulation of the pesticides chlorpyrifos and difenoconazole and their mixtures in radish plants grown in soils contaminated with microplastics was investigated by Ju et al. (2024) [76]. The microplastics used were pellets of LDPE and a biopolymer (mixture of 85% PBAT, 10% PLA and 5% calcium carbonate), with sizes ranging from 200 to 500 µm. The experiment involved growing radish seeds in pots with different treatments and exposure conditions. The pesticide mixture treatments had contaminant concentrations of 0.2% (*w*/*w*) and 15 mg/Kg for each pesticide. The germination of the radish seeds was measured. Pesticide residues in soil and radish and their distribution in the plant were sampled after five weeks of incubation. The study results indicate that germination was not affected by any treatment, and the growth effects were primarily caused by the presence of microplastics rather than the pesticide. The study found that the aged microplastics, with or without pesticides, had a negative impact on radish root biomass. Furthermore, the aged microplastics resulted in higher chlorpyrifos bioaccumulation in radish roots compared to virgin microplastics when the soil was contaminated with a single chlorpyrifos. However, chlorpyrifos bioaccumulation in the radish roots was higher than in the soil contaminated with single pesticides and LDPE when the soil was contaminated with pesticide/LDPE mixtures, causing a synergistic effect on the plant.

3. Invertebrates

Sun et al. (2021) [77] investigated the combined effects of microplastics and the pesticide dufulin on insect bioaccumulation, oxidative stress and metabolic profiles in earthworms (*Eisenia fetida*). The study involved different treatments, with the dufulin concentration fixed at 10 mg/Kg and the microplastic concentration varying between high (3000 mg/Kg) and low (300 mg/Kg). The bioaccumulation test lasted 28 days, while the toxicity test lasted 14 days. After both exposure periods, it was observed that the presence of microplastics increased the bioaccumulation of pesticides when combined, and microplastics caused oxidative stress to insects, as evidenced by the increased lipid peroxidation levels after 7 days of exposure to the high concentration of MP and dufulin and increased superoxide dismutase activity after 14 days. The metabolism analysis using 1H NMR revealed that exposure to dufulin significantly altered the relative abundance of 14 metabolites and 2 pathways, whereas exposure to both dufulin and microplastics significantly altered the relative abundance of 21 metabolites and 3 pathways. The mixture of contaminants was more toxic than the individual exposure, causing a synergistic effect.

In Boughattas et al. (2024) [78], single and co-exposure to the pesticide 2,4-dichlorophenoxyacetic acid (2,4-D) MP at concentrations of 7 mg/Kg and 10 µg/Kg for 7 and 14 days, respectively, were used to study changes in the metabolome and microbial structure in the intestine of *Eisenia andrei*. The results showed that worms ingested microplastics and that the pesticide bioaccumulation was greater in the presence of plastics. The gut microbial diversity of bacteria and archaea was assessed and showed significant differences from the controls. Several metabolic pathways, including energy metabolism, protein homeostasis and the inflammatory system, were negatively affected by exposure to this mixture, generating stress in the worms. So, the interaction seems to be synergistic.

Lu et al. (2023) [79] investigated the toxic effects of polystyrene microplastics (100 µm) and the antifungal drug ketoconazole, as well as their bioaccumulation, in the benthic organism *Limnodrilus hoffmeistteri*. The experiment involved exposing the organism to different concentrations of microplastics and pesticides (0.1, 1, 10 and 100 µg/L). Treatments included control, ketoconazole, microplastics and a combination of both in a simulated benthic environment. The organisms were exposed to the different treatments for 28 days, and the results on bioaccumulation, behavior and oxidative stress were obtained. Pesticide accumulation increased significantly in the presence of microplastics. Both pollutants, individually and together, caused a decreased weight of parents and progeny, inflammation, behavioral changes and oxidative stress. The mixture of pollutants seemingly had a synergistic effect on the parental generation.

The study of the interactions between microplastics and different compounds (pharmaceutical, industrial and agrochemical) in terrestrial environments reveals a complex and often synergistic detrimental effect. The combination of pollutants has been observed to result in a deterioration of soil quality, a reduction in microbial diversity and an alteration of nutrient uptake in plants. In invertebrates, the combined effects of contaminants on behavior, health and gut microbiota have been observed to increase antibiotic resistance. In plants, the size of the microplastics and the combination with industrial compounds has been observed to influence growth and enzyme activity, with effects that can be both synergistic and antagonistic. In vertebrates, co-exposure to nanoparticles and microplastics has been observed to result in developmental and survival challenges, including increases in morphological abnormalities. Studies investigating interactions between agrochemical compounds and microplastics have revealed that these interactions can vary, affecting nutrient cycling and plant growth in ways that are more deleterious to invertebrates. These findings underscore the intricate and multifaceted nature of contaminant interactions, underscoring the necessity to assess and understand them in a more nuanced and comprehensive manner to effectively mitigate their adverse environmental impacts.

### 3.3. Summary of MPs and NPs in Aquatic and Terrestrial Environments

The information presented in Table 1 and Table 2 is a summary of data from the articles reviewed in the text, organized according to aquatic and terrestrial environments, respectively. It takes into consideration the type of polymer, the microcontaminants themselves and the biological effects. 

In the numerous studies reviewed, polystyrene has been recognized as a prevalent material utilized in investigations involving micro and nanoplastics. Frequently utilized as a model microplastic in toxicological research due to its residual environmental content, polystyrene is an important subject in this field of study [80,81]. Only a limited number of studies have successfully quantified the effective concentration 50 (EC50) of the mixture or constructed a comprehensive dose–response curve for the documented ecotoxicological endpoints. Although some EC50 values have been reported for mixtures in aquatic environments, no interaction determination has been made about the reported EC50 calculations for individual compounds in terrestrial environments. The reviewed articles reveal a notable absence of research exploring the interactions between pollutants. Many of these interactions are based on the effects caused by the combination of pollutants, without calculating any toxicological parameters.

**Figure 2:**
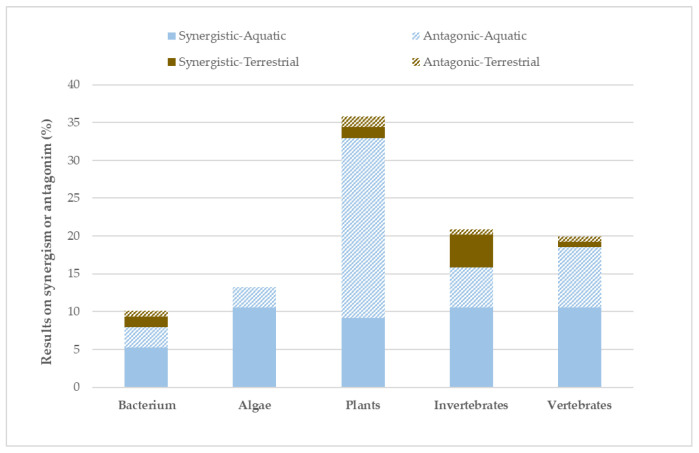
Combined effect observed in aquatic and terrestrial organisms for mixtures of MPs and NPs including emerging pollutants. The patterns indicate antagonism, and the plain colors denote synergistic effect.

Figure 2 was created from the collected data presented in Table 1 and Table 2. For mixtures of microplastics or nanoplastics with emerging contaminants, this figure shows that the percentage of observations pertaining to the specific types of interactions across all studies consulted has been calculated. The information collected is grouped by organism type and shows that 25% of the observations were made for aquatic and terrestrial invertebrates, approximately equal. Vertebrates (mainly aquatic) are the next most studied group of organisms. This is followed by plants, where most observations were also made in aquatic environments. For algae and bacteria, the proportion of ecotoxicological results reported for mixtures was lower. Furthermore, aquatic organisms accounted for most combined-effect studies.

## 4. Conclusions and Future Predictions

The combined effect of microplastics or nanoplastics on emerging contaminants has been the subject of greater scrutiny in aquatic environments, where EC50 values for individual compounds have been documented. A small number of studies have reported combined values to ascertain the nature of the interaction between micropollutants. The values in question exhibit considerable variability, contingent on the specific type of polymer, its dimensions, the emerging contaminant under examination, and the organism employed in the study. Our analysis indicates that, in aquatic environments, synergistic effects are more prevalent than antagonistic effects. This suggests that microplastics intensify the impact of other pollutants in a mixture. The joint toxicological action of microplastics or nanoplastics with emerging contaminants has been less extensively investigated in terrestrial environments.

Nevertheless, an alternative interpretation is that synergy may in fact be the predominant phenomenon, as evidenced by the effects observed in different species. It would be advantageous for future research on ecotoxicity to place a greater emphasis on the utilization of toxicological modeling for the investigation of mixtures. Risk assessments based on single contaminants may underestimate the true magnitude of the problem due to the evidence of synergistic and antagonistic effects. Furthermore, the variability in responses between organisms and contaminant types highlights the complexity of chemical interactions in ecosystems and the gaps in knowledge regarding this issue. To develop effective mitigation strategies and appropriate environmental policies, it is essential that future research focuses on a wider range of contaminant combinations and at different levels of the food chain.

## Figures and Tables

**Table 1:** Summary of aquatic environments: classification by organisms, type, size and concentration of plastics and micropollutants tested in the studies. Biological responses analyzed, EC50 for individual and combined exposures and the effect of the mixture can be observed.

Organisms		MNPs	Pollutant Dose	Exposure Time (Days)	Biological Response	EC50 Individual	EC50 Combined	Effect	References
Bacterium	*Microcystis* *aeruginosa*	PS (ii), amino-modified PS (i) and PS-COOH (ii)	Boron	4	Growth rate (A), chlorophyll a content, oxidative stress, cellular structure and microcystines	(A) Boron: 26.6 mg/L	NA	(i) Synergistic; (ii) additive	[45]
		NP-PS (amino modified)	Glyphosate	4	Chlorophyll a content (growth rate)	Glyphosate 48 h: 8.1 mg/L; glyphosate 96 h: 6.3 mg/L	NA	Antagonistic	[57]
	*Anabaena* sp. *PCC7120*	PS, PET, PLA and POM (1 g/20 mL) virgin and aged	Azithromycin (500 µg/L) and Clarithromycin (1000 µg/L)	3	Chlorophyll a Growth rate	NA	NA	Synergistic	[33]
Algae	*Chlorella pyrenoids*	PS 5 µm (50 mg/L)	Amphetamine (0.001, 0.1, 1 and 5 mg/L)	4	Concentration photosynthetic pigments, growth rate (A), SOD and MDA activities	(A) 0.45 ppm	(A) 0.67 ppm	Synergistic	[34]
	*Tetraselmis chuii*	MP 1–5 µm (1.5 mg/L)	Procainamide and doxycycline (4, 8, 16, 32, 64, 128 and 256 mg/L)	4	Chlorophyll content (A); growth rate (B)	Procainamide: (A) 143 mg/L, (B) 104 mg/L; doxycycline: (A) 14 mg/L, (B) 22 mg/L	Procainamide + MP: (A) 31 mg/L, (B) 125 mg/L; doxycycline + MP: (A) 7 mg/L, (B) 11 mg/L	Synergistic	[35]
		MP fluorescent red (0.3, 0.9 and 4 mg/L)	Gold nanoparticles (0.1, 0.3 and 3 mg/L)	4	Growth rate	NA	NA	Synergistic	[46]
	*Chlorella vulgaris*	PP (ii), PS (i), PVC (i) (0, 0.1, 0.2, 0.3, 0.4 and 1 g/L)	Pb, Cu, Cr and Cd (0, 50, 500 and 1000 µg/L)	4	Growth rate (i, ii) oxidative stress	NA	NA	(i) Antagonistic; (ii) synergistic	[44]
Plants	*Spirodela polyhriza*	NP-PS (12.5, 25, 50, 100 and 200 mg/L)	Wastewater (1:16, 1:8, 1:4, 1:2 and 1:1)	3	Growth inhibition (A) chlorophyll fluorescence	(A) NP-PS: 170 + −14 mg/L; WW: 0.94 + −0.27 mg/L	NA	Antagonistic	[36]
		NP-PS 80 nm (0.5, 5, 10 and 20 mg/L)	PCB-52 (0.1 mg/L)	10	Growth rate, photosynthetic pigments, osmoregulation and antioxidant response	NA	NA	Synergistic	[47]
	*Hydrilla verticillata*	NP-PS (1 and 10 mg/L)	Bisphenol F (10 mg/L)	16	Growth rate, chlorphylls a and b content and antioxidant response	NA	NA	Antagonistic	[48]
	*Myriophyllum virticillatum* L.	NP-PS 100 nm (0, 10 and 50 mg/L)	As (0 and 0.1 (i) and 1 (ii) mg/L)	24	Growth rate, oxidative stress, osmotic regulation and metabolic response	NA	NA	(i) Synergistic; (ii) antagonistic	[49]
	*Spirodela polyrhiza*	MP PS (10–50 µm) 100 mg/L	Ciprofloxacin 2 mg/L	15	(i) Specific leaf area, (ii) chlorophylls a and b, (iii) MDA, (iv) catalase activity and (v) soluble sugar content	NA	NA	(i) Additivity/synergistic; (ii) antagonistic; (iii) antagonistic/additive; (iv) antagonistic; (v) antagonistic/additive	[37]
	*Lemna minor*					NA	NA	(i) Additive; (ii) additive; (iii) antagonistic; (iv) antagonistic; (v) synergistic/antagonistic	
	*Hydrilla verticillata*	MP PS (i) 5 µm, (ii) 50 µm and (iii) 500 µm) 75 mg/L	Tetracycline 50 mg/L	35	Total length, specific leaf area, branch net, fresh weight, chlorophylls a and b, soluble sugar, MDA and catalase activities	NA	NA	(i) Synergistic/antagonistic; (ii) antagonistic; (iii) antagonistic	[38]
	*Elodea nuttallii*					NA	NA	(i) Synergistic/antagonistic; (ii) antagonistic; (iii) antagonistic	
	*Cyperus papyrus* L.	NP (A) amino-modified PS and (B) PS-COOH 50 mg/L	Imidacloprid 1.0 mg/L	28	Growth inhibition, chlorophyll content, MDA, and antioxidant enzyme activities	NA	NA	Synergistic	[58]
	*Ceratophyllum demersum* L.	NP-PS 100 nm (5 and 10 mg/L)	Cd (0.1, 0.5 and 1 mg/L)	14	Fresh weight, chlorophyll content and antioxidant response	NA	NA	Synergistic	[50]
Invertebrates	*Ceriodaphnia dubia*	PS 1 µm	Acyclovir and Imidacloprid	1 to 7	Reproduction (i) and genotoxicity (ii)	PS: 1.68 µg/L Acyclovir: 0.04 µg/L Imidacloprid: 1358 µg/L	NA	(i) Additive; (ii) antagonistic	[40]
	*Cassotrea brasiliana*	Environmental MP (250 mg/L)	Triclosan	3 to 7	Tissue biomarkers	NA	NA	Additive	[39]
	*Chironomus tepperi*	PE 10–27 µm (5 mg/L)	Bifenthrin (range 0.1–3.2 μg/L)	2	Survival rate	Synthetic water (i) EC50: 0.5 µg/L; River water (ii) EC50: 1.3 µg/L	Synthetic water (i) EC50: 1.3 µg/L; River water (ii) EC50: 1.4 µg/L	(i) Antagonistic; (ii) synergistic	[59]
	*Daphnia magna*	PET + PA and PE	Glyphosate	7	Survival rate	NA	NA	Not clear	[60]
		PS 70 nm, 1 and 5 µm (0, 30, 50, 70, 90 and 120 mg/L)	Carbamazepine (0, 5, 10, 15, 20, 25 and 30 mg/L)	2 to 21	Reproduction	700 nm: 24.15; 1 µm:14.09; 5 µm: 21.66	700 nm + 50 mg/L CBZ: 10.69; 1 µm + 50 mg/L CBZ: 15.60; 5 µm + 50 mg/L CBZ: 14.56; CBZ + 5 mg/L 700 nm MP-PS: 74.01; CBZ + 5 mg/L 1 µm MP-PS: 82.12; CBZ + 5 mg/L 5 µm MP-PS: 50.08	Synergistic	[41]
		PE 1–4 µm (0, 1 and 10 mg/L)	Deltamethrin (0, 40 ng/L)	21	Survival rate (A), atching and body shape	(A) EC50 24 h MP 1–10 and DM40: 0.815 and 0.855 mg/L 0.873 ng/L EC50 48 h MP 1–10 and DM40: 0.641 and 0.600 mg/L, 0.575 ng/L	NA	Synergistic	[61]
	*Pomeacea paludosa*	PP (10 µg/L)	ZnO nanoparticles (10 µg/L)	28	Behavioral parameters, antioxidant, digestive, and neurotransmission enzymes	NA	NA	Synergistic	[51]
Vertebrates	*Cairina moschata*	MP (1000 µg/L)	Chlorotetracycline (50 mg/Kg)	56	Inflammation bioaccumulation	NA	NA	Antagonistic	[42]
	*Oncorhynchus mykiss*	HDPE (0, 1000 and 2000 mg/Kg)	Enrofloxacin (0, 1.35 and 2.7 mL/Kg)	21	Blood biomarkersand oxidative stress	NA	NA	Synergistic	[43]
	*Ctenopharyngodon idella*	NP-PS 20–26 nm (760 µg/L)	ZnO nanoparticles (760 µg/L)	72	Genotoxicity, oxidative stress and behavioral test	NA	NA	Not clear	[53]
	*Danio rerio*	PS 5–50 µm (10 mg/L)	3,6dibromocarbazole (0.5 mg/L)	4	Oxidative stress	NA	NA	Antagonistic	[54]
		PS (3 mg/L)	Phenanthrene (0.2 mg/L)	1 to 24	Accumulation, antioxidant enzymes, gene expression and gut microbiota	NA	NA	Synergistic	[55]
		PS (20 µg/L)	Imidacloprid (100 µg/L)	21	Inflammatory parameters, oxidative stress and glycolytic metabolism parameters	NA	NA	Synergistic	[62]
	*Oryzias melastigma*	PS 5–50 µm (100 µg/L)	Cd (10 µg/L), Pb (50 µg/L) and Zn (100 µg/L)	30	Microbial community gonadal development	NA	NA	Not observed	[52]
	*Oreochromus niloticus*	PE (10 mg/L)	Up Grade^®^ (2.92 mg/L)	4	(A) Mortality hematological and biochemical parameters	(A) LD50 (48 h) 56.37; LD50 (72 h) 36.10; LD50 (96 h) 29.16	NA	Antagonistic	[63]
		NP-PS 50 nm (5 mg/L)	Engine oil (1%)	15	Hematological parameters, oxidative stress and inflammation	NA	NA	Synergistic	[56]

(A, B) Parameter to calculate the EC50. (i, ii, iii…) More than one interaction condition observed.

**Table 2:** Summary of terrestrial environments: classification by organisms, type, size and concentration of plastics and micropollutants tested in the studies. Biological responses analyzed, EC50 for individual and combined exposures and the effect of the mixture can be observed.

Organisms		MNPs	Pollutant Dose	Exposure Time (Days)	Biological Response	EC50 Individual	EC50 Combined	Effect	References
Bacterium	Microbial comunities of soils	PE (1% *w*/*w*)	Ciprofloxacin (10 mg/Kg)	35	16s-RNA secuenciation and total nitrogen	NA	NA	Synergistic	[64]
		PE (5% *w*/*w*)	Tetracycline (10 mg/Kg)	35	16s-RNA secuenciation, ARGs and enzymes	NA	NA	Synergistic	[65]
		MP aged mulch films (2 wt/2 wt)	Imidacloprid (0.5 and 5 mg/Kg) and flumioxazin (0.3 and 3 mg/Kg)	56	16s-RNA secuenciation and elemental analysis	NA	NA	Antagonistic	[74]
Plants	*Chrysanthemum coronarium* L.	PS 1000–500 nm (4% *w*/*w*)	Norfloxacin (50 mg/Kg) and sulfadiazine (10 mg/Kg)	60	Acummulation, elemental analysis and cellular distribution	NA	NA	Synergistic	[66]
	Tomato plant	PS-COO- (i), PS, PS-NH3+ (2 mg/L)	Dufulin (50 mg/L)	60	Biodisponibility	NA	NA	Antagonistic (i)	[75]
	Wheat	PP 50 (i) and 100 (ii) µm (500 mg/L)	Cadmium (40 mg/L)	3 and 7	Germination, morphology and oxidative stress	NA	NA	(i) Antagonistic; (ii) synergistic	[70]
	Radish plant	LDPE and biopolimer, pristine and aged (i), 200–500 µm (0.2% *w*/*w*)	Chlorpyrifos and difenoconazole (15 mg/Kg)	35	Growth rate	NA	NA	(i) Synergistic	[76]
Invertebrates	*Hediste diversicolor*	PET 125 µm–1 mm (0.032 and 0.054 g/L)	Ciprofloxacin (130 ng/L and 1300 ng/L)	28	Behavioral parameters, metabolic enzymes and oxidative stress	NA	NA	Synergistic	[67]
	*Enchytraeus crypticus*	PA and PVC (1000 mg/Kg)	Tetracycline (20 mg/Kg)	21	Microbial community and analysis of antibiotic resistant genes	NA	NA	Not observed	[68]
	*Folsomia candida*	NP (0.015 and 600 mg/Kg)	BPA and diphenhydramine (0, 1, 10, 100 and 2000 mg/Kg)	28	(i) Reproduction; (ii) lipid peroxidation	BPA 1399.3 mg/Kg, DPH 1498.2 mg/Kg	NA	(i) Synergistic; (ii) antagonistic	[71]
		PS 2 µm (1% *w*/*w*)	Sulfamethoxazole (1 mg/L)	28	Microbial community, analysis of antibiotic resistant genes and C:N factionation	NA	NA	Synergistic	[69]
	*Eisenia fetida*	MP	Dufulin (10 mg/Kg)	14	Bioacumulation, oxidative stress and metabolic profile	NA	NA	Synergistic	[77]
	*Eisenia andrei*	Environmental MP (PE, PP, PET, PEVA and PA) (10 µg/Kg)	2,4-dochlorophenoxyacetic acid (7 mg/Kg)	14	Microbial community and metabolonics	NA	NA	Synergistic	[78]
	*Limnodrilus hoffmeistteri*	PS 10 µm (0.1, 1, 10 and 100 µg/g)	Ketoconazole (0.1, 1, 10 and 100 µg/g)	28	Reproductive, behavioral parameters and oxidative stress	NA	NA	Synergistic	[79]
	*Pontastacus leptodactylus*	PE 15–25 µm (0, 0.5 and 1 mg/L)	CuSO_4_ (0.5 and 1 mg/L)	28	Hemolymph parameters and oxidative stress	NA	NA	Synergistic	[72]
Vertebrates	*Aquarana catesbeiana*	PE 35.4 µm (10 µg/L)	TiO_2_ nanoparticles (10 µm/L)	4	Survival rate and hatching rate	NA	NA	Antagonistic	[73]

(i, ii) More than one interaction condition observed.

## Data Availability

The data presented in this study are available on request from the corresponding author.

## Supplementary Materials

The following supporting information can be downloaded at: https://www.mdpi.com/article/10.3390/toxics12080589/s1, Table S1: Number of published works on microplastics and nanoplastics in the period 2017–2023 classified by research areas; Table S2. Number of published works on microplastics and nanoplastics plus pharmaceuticals contaminants the period 2017–2023, classified by research areas; Table S3: Number of published works on microplastics and nanoplastics plus pesticides contaminants the period 2017–2023, classified by research areas; Table S4: Interannual rate of increment of publications and citations on articles including microplastics, nanoplastics, pharmaceuticals or pesticides in the period 2017–2023. The calculated values of increment in citation are shown in brackets.
