# Supplementary material for: Mixtures of Micro and Nanoplastics and Contaminants of Emerging Concern in Environment: What We Know about Their Toxicological Effects
Source: Toxics. 2024 Aug 13;12(8):589. doi: 10.3390/toxics12080589 (PMC11359687; doi:10.3390/toxics12080589)
Supplement: Supplementary file 1 [file toxics-12-00589-s001.zip › toxics-3105261-supplementary.pdf]

Supplementary Materials

The following tables present the data collected on the number of published papers referring to microplastics and nanoplastics and their mixture with other micropollutants such as pharmaceuticals and pesticides. The reference period for this review work is 2017 to 2024, but in this tables, we only include numeric results grouped by research areas for the period 2017-2023. This was done to determine the interannual rate of increment of publications and citations, when the inclusion of the published work of 2024 can disturb the profile obtained due to 2024 is not yet complete. The total number of papers referring to microplastics and nanoplastics is 3315, of which 115 also include pharmaceuticals and 102 pesticides. All tables show the distribution of these papers by knowledge area.

Table S1. Number of published works on microplastics and nanoplastics in the period 2017-2023, classified by research areas

**Research Areas**	**Record Count**	**% of 3,315**
Environmental Sciences Ecology	2,831	85.40%
Public Environmental Occupational Health	2,176	65.64%
Toxicology	2,113	63.74%
Science Technology Other Topics	1,224	36.92%
Biochemistry Molecular Biology	1,033	31.16%
Engineering	962	29.02%
Water Resources	890	26.85%
Materials Science	816	24.62%
Zoology	768	23.17%
Chemistry	760	22.93%
Marine Freshwater Biology	737	22.23%
Energy Fuels	546	16.47%
Agriculture	512	15.45%
Pharmacology Pharmacy	506	15.26%
Genetics Heredity	488	14.72%
Business Economics	405	12.22%
Physiology	379	11.43%
Cell Biology	343	10.35%
Plant Sciences	330	9.96%
Physics	286	8.63%
Food Science Technology	266	8.02%
Gastroenterology Hepatology	254	7.66%
Microbiology	249	7.51%
Developmental Biology	237	7.15%
Biotechnology Applied Microbiology	224	6.76%
Instruments Instrumentation	220	6.64%
Reproductive Biology	212	6.40%
Nutrition Dietetics	180	5.43%
Immunology	175	5.28%
Pathology	174	5.25%
Biophysics	171	5.16%
Endocrinology Metabolism	160	4.83%
Spectroscopy	159	4.80%
Anatomy Morphology	158	4.77%

Infectious Diseases	142	4.28%
Neurosciences Neurology	123	3.71%
Mathematical Computational Biology	119	3.59%
Computer Science	117	3.53%
Biodiversity Conservation	107	3.23%
Meteorology Atmospheric Sciences	102	3.08%
Veterinary Sciences	102	3.08%
Behavioral Sciences	99	2.99%
Mathematics	83	2.50%
Cardiovascular System Cardiology	77	2.32%
Radiology Nuclear Medicine Medical Imaging	75	2.26%
Oceanography	74	2.23%
Polymer Science	74	2.23%
Respiratory System	71	2.14%
Fisheries	69	2.08%
Education Educational Research	65	1.96%
Hematology	50	1.51%
Life Sciences Biomedicine Other Topics	46	1.39%
Crystallography	44	1.33%
Geology	36	1.09%
Health Care Sciences Services	35	1.06%
Electrochemistry	26	0.78%
Oncology	26	0.78%
Dermatology	25	0.75%
Geography	25	0.75%
Entomology	22	0.66%
Microscopy	22	0.66%
Urology Nephrology	22	0.66%
Geochemistry Geophysics	21	0.63%
Geriatrics Gerontology	21	0.63%
Information Science Library Science	21	0.63%
Obstetrics Gynecology	20	0.60%
Medical Laboratory Technology	19	0.57%
Pediatrics	17	0.51%
Anthropology	15	0.45%
General Internal Medicine	15	0.45%
Optics	15	0.45%
Physical Sciences Other Topics	14	0.42%
Research Experimental Medicine	13	0.39%
Forestry	12	0.36%
Evolutionary Biology	11	0.33%
Mycology	11	0.33%
Thermodynamics	11	0.33%
Psychology	10	0.30%
Social Issues	9	0.27%
Social Sciences Other Topics	9	0.27%
Automation Control Systems	8	0.24%
Communication	8	0.24%
Government Law	8	0.24%
Sport Sciences	8	0.24%
Dentistry Oral Surgery Medicine	5	0.15%

Parasitology	5	0.15%
Acoustics	4	0.12%
Demography	4	0.12%
Mechanics	4	0.12%
Metallurgy Metallurgical Engineering	4	0.12%
Psychiatry	4	0.12%
Sociology	4	0.12%
Surgery	4	0.12%
Arts Humanities Other Topics	3	0.09%
Medical Informatics	3	0.09%
Allergy	2	0.06%
Anesthesiology	2	0.06%
Construction Building Technology	2	0.06%
Nuclear Science Technology	2	0.06%
Ophthalmology	2	0.06%
Physical Geography	2	0.06%
Public Administration	2	0.06%
Telecommunications	2	0.06%
Transplantation	2	0.06%
Virology	2	0.06%
Architecture	1	0.03%
Art	1	0.03%
Film Radio Television	1	0.03%
International Relations	1	0.03%
Legal Medicine	1	0.03%
Mineralogy	1	0.03%
Orthopedics	1	0.03%
Remote Sensing	1	0.03%
Robotics	1	0.03%
Theater	1	0.03%
Urban Studies	1	0.03%

Table S2. Number of published works on microplastics and nanoplastics plus pharmaceuticals contaminants the period 2017-2023, classified by research areas

Research Areas	Record Count	% of 102
Environmental Sciences Ecology	89	87.26%
Toxicology	80	78.43%
Public Environmental Occupational Health	72	70.59%
Pharmacology Pharmacy	55	53.92%
Biochemistry Molecular Biology	37	36.28%
Chemistry	36	35.29%
Science Technology Other Topics	35	34.31%
Water Resources	33	32.35%
Zoology	24	23.53%
Marine Freshwater Biology	23	22.55%
Engineering	21	20.59%
Genetics Heredity	19	18.63%

Materials Science	19	18.63%
Agriculture	18	17.65%
Pathology	16	15.69%
Energy Fuels	15	14.71%
Gastroenterology Hepatology	14	13.73%
Cell Biology	13	12.75%
Business Economics	11	10.78%
Food Science Technology	11	10.78%
Physiology	10	9.80%
Immunology	9	8.82%
Infectious Diseases	9	8.82%
Plant Sciences	9	8.82%
Reproductive Biology	9	8.82%
Endocrinology Metabolism	8	7.84%
Biotechnology Applied Microbiology	6	5.88%
Nutrition Dietetics	6	5.88%
Biophysics	5	4.90%
Instruments Instrumentation	5	4.90%
Microbiology	5	4.90%
Radiology Nuclear Medicine Medical Imaging	5	4.90%
Respiratory System	5	4.90%
Meteorology Atmospheric Sciences	4	3.92%
Neurosciences Neurology	4	3.92%
Veterinary Sciences	4	3.92%
Behavioral Sciences	3	2.94%
Developmental Biology	3	2.94%
Mathematical Computational Biology	3	2.94%
Oncology	3	2.94%
Physics	3	2.94%
Spectroscopy	3	2.94%
Computer Science	2	1.96%
Geriatrics Gerontology	2	1.96%
Life Sciences Biomedicine Other Topics	2	1.96%
Mathematics	2	1.96%
Obstetrics Gynecology	2	1.96%
Urology Nephrology	2	1.96%
Anatomy Morphology	1	0.98%
Anesthesiology	1	0.98%
Arts Humanities Other Topics	1	0.98%
Biodiversity Conservation	1	0.98%
Dentistry Oral Surgery Medicine	1	0.98%
Dermatology	1	0.98%
Education Educational Research	1	0.98%
Entomology	1	0.98%
Fisheries	1	0.98%
Forestry	1	0.98%
General Internal Medicine	1	0.98%
Health Care Sciences Services	1	0.98%
Microscopy	1	0.98%
Ophthalmology	1	0.98%
Polymer Science	1	0.98%

Psychology	1	0.98%
Social Issues	1	0.98%

Table S3. Number of published works on microplastics and nanoplastics plus pesticides contaminants the period 2017-2023, classified by research areas

**Research Areas**	**Record Count**	**% of 115**
Environmental Sciences Ecology	108	93.91%
Chemistry	93	80.87%
Toxicology	91	79.13%
Public Environmental Occupational Health	87	75.65%
Water Resources	57	49.57%
Marine Freshwater Biology	45	39.13%
Biochemistry Molecular Biology	44	38.26%
Zoology	38	33.04%
Pharmacology Pharmacy	36	31.30%
Agriculture	34	29.57%
Genetics Heredity	34	29.57%
Science Technology Other Topics	29	25.22%
Microbiology	24	20.87%
Engineering	22	19.13%
Infectious Diseases	22	19.13%
Physiology	22	19.13%
Plant Sciences	22	19.13%
Energy Fuels	17	14.78%
Business Economics	16	13.91%
Materials Science	15	13.04%
Biotechnology Applied Microbiology	11	9.57%
Developmental Biology	10	8.70%
Food Science Technology	10	8.70%
Reproductive Biology	10	8.70%
Pathology	8	6.96%
Nutrition Dietetics	7	6.09%
Anatomy Morphology	6	5.22%
Biodiversity Conservation	6	5.22%
Cell Biology	6	5.22%
Behavioral Sciences	5	4.35%
Gastroenterology Hepatology	5	4.35%
Instruments Instrumentation	5	4.35%
Neurosciences Neurology	5	4.35%
Immunology	4	3.48%

Mathematical Computational Biology	4	3.48%
Veterinary Sciences	4	3.48%
Computer Science	3	2.61%
Endocrinology Metabolism	3	2.61%
Entomology	3	2.61%
Physics	3	2.61%
Respiratory System	3	2.61%
Biophysics	2	1.74%
Cardiovascular System Cardiology	2	1.74%
Education Educational Research	2	1.74%
Fisheries	2	1.74%
Health Care Sciences Services	2	1.74%
Oncology	2	1.74%
Spectroscopy	2	1.74%
Anthropology	1	0.87%
Dermatology	1	0.87%
Evolutionary Biology	1	0.87%
Forestry	1	0.87%
Life Sciences Biomedicine Other Topics	1	0.87%
Mathematics	1	0.87%
Medical Laboratory Technology	1	0.87%
Meteorology Atmospheric Sciences	1	0.87%
Obstetrics Gynecology	1	0.87%
Oceanography	1	0.87%
Ophthalmology	1	0.87%
Pediatrics	1	0.87%
Radiology Nuclear Medicine Medical Imaging	1	0.87%
Social Issues	1	0.87%
Thermodynamics	1	0.87%

Table S4. Interannual rate of increment of publications and citations on articles including microplastics, nanoplastics, pharmaceuticals or pesticides in the period 2017-2023. The calculated values of increment in citation are shown in brackets.

Period	MNPs (%)	MNPs + pharmaceuticals (%)	MNPs + pesticides (%)
2023-2022	18 (30)	28 (25)	-3 (31)
2022-2021	31 (41)	38 (37)	55 (42)
2021-2020	51 (64)	44 (68)	35 (71)
2020-2019	49 (66)	67 (56)	73 (61)
2019-20218	63 (76)	0 (77)	67 (89)
2018-2017	56(94)	67 (100)	0 (0)
